# Rachitisme patent révélé par la perte du contrôle des convulsions chez un garçon infirme moteur cérébral

**DOI:** 10.11604/pamj.2013.14.25.896

**Published:** 2013-01-16

**Authors:** Hubert Désiré Mbassi Awa, David Chelo, Mina Njiki Kinkela, Paul Olivier Koki Ndombo

**Affiliations:** 1 Centre Mère et Enfant de la Fondation Chantal BIYA, Cameroun; 2 Faculté de Médecine et de Sciences Biomédicales de l'Université de Yaoundé I, Cameroun

**Keywords:** Rachitisme, infirmité motrice cérébrale, épilepsie, vitamine D, phénobarbital, rickets, Cerebral Palsy, epilepsy, vitamin D, phenobarbital

## Abstract

La perte du contrôle des crises chez un patient compliant au traitement est toujours source de préoccupations diagnostiques, thérapeutiques et pronostiques. Nous rapportons un cas de rachitisme chez un patient de 4 ans, infirme moteur cérébral et épileptique sous traitement par phénobarbital depuis 2 ans; rachitisme découvert à la faveur d'une perte du contrôle épileptique. Le patient était admis pour des convulsions répétées en contexte afébrile. L'observance thérapeutique était bonne, et aucune convulsion n'avait été observée pendant les 12 mois précédents. Il ne recevait pas de vitamine D. Le rachitisme était suspecté cliniquement, et confirmé par les trouvailles radiologiques et biologiques. Le contrôle des crises était retrouvé dès le 3ème jour d'hospitalisation après apports de calcium intraveineux et de vitamine D. Les convulsions étaient imputées à une hypocalcémie sur rachitisme. La prise prolongée de phénobarbital sans supplémentation en vitamine D, ainsi qu'une exposition solaire insuffisante étaient incriminées. Avant toute escalade thérapeutique, des convulsions hypocalcémiques et un rachitisme doivent toujours être exclus devant une perte du contrôle des crises chez tout patient épileptique à mobilité réduite. Par ailleurs, une supplémentation en vitamine D et une exposition suffisante au soleil devront être recommandées pour certains traitements antiépileptiques au long cours.

## Introduction

L’épilepsie est une maladie neurologique chronique, nécessitant généralement un traitement au long cours. Un contrôle complet des crises, une absence d'effets secondaires des médicaments, et l'amélioration de la qualité de vie du patient sont des impératifs du traitement. La reprise des crises chez un patient compliant sous antiépileptique est généralement source de préoccupations d'ordres diagnostique, thérapeutique et pronostique. Une anamnèse et une recherche étiologique minutieuses sont cruciales pour élucider la situation.

Nous rapportons un cas de rachitisme chez un enfant infirme moteur cérébral et épileptique, sous traitement prolongé par phénobarbital. Le patient avait requis un avis médical pour une perte du contrôle épileptique. Nous soulignons la nécessité d'exclure des convulsions occasionnelles dans ces cas avant toute modification ou escalade thérapeutique.

## Patient et observation

Un patient de 4 ans était hospitalisé pour des convulsions répétées, en contexte afébrile. L'anamnèse retrouvait une naissance à terme en contexte d'asphyxie périnatale sévère et prolongée; un retard psychomoteur global sur un tableau d'infirmité motrice cérébrale constitué dès le deuxième semestre de vie; une épilepsie apparue au début de la deuxième année de vie, initialement traitée par du valproate de sodium, puis la carbamazépine. L'observance à ces deux médications était mauvaise en raison de leur coût prohibitif. Depuis deux ans, l'enfant était sous phénobarbital (Gardenal^®^) avec une bonne observance, et son épilepsie était sous contrôle depuis près de 12 mois. Il n'y avait pas eu de passage au phénobarbital générique. Aucune supplémentation en vitamine D n’était prescrite. On ne retrouvait pas d'antécédents de comitialité dans la famille. Les parents déclaraient une alimentation équilibrée, comportant des produits laitiers.

A l'examen clinique, le patient présentait une microcéphalie à 44,5 cm (- 4 DS). Il n'y avait pas de signes d'irritation méningée. Le poids était de 12,2 kg (< 3^e^ percentile pour l’âge). L'examen retrouvait par ailleurs une tuméfaction médio-claviculaire gauche suggestive de cal osseux sur une fracture antérieure ; des bourrelets épiphysaires aux poignets, un chapelet costal. On notait une hypotonie axiale, une hypertonie spastique des membres inférieurs avec varus équin, mais sans déformations. Les hanches et la position assise étaient stables, les réactions de protection étaient présentes. Le patient se déplaçait à 4 pattes avec quelques difficultés, il était coté au niveau IV de la classification de la fonction motrice globale selon Palisano [1]. Il associait quelques mots.

Le diagnostic de rachitisme avec convulsions hypocalcémiques était évoqué. Mais une pharmaco-résistance secondaire sur une encéphalopathie épileptogène restait envisageable. L’éventualité d'une maltraitance était possible.

La biologie sanguine montrait: une calcémie à 1,70 mmol/L (N: 2,2 - 2,70 mmol/L), une magnésémie à 0,67 mmol/L (N: 0,60 - 0,95 mmol/L), une phosphorémie à 1,22 mmol/L (N: 1,20 - 1,80 mmol/L), des phosphatases alcalines à 887 UI/L (N: 145 - 420 UI/L), des fonctions hépatique et rénale normales. Les dosages de la vitamine D et de la parathormone n’étaient pas disponibles.

**Figure 1:**
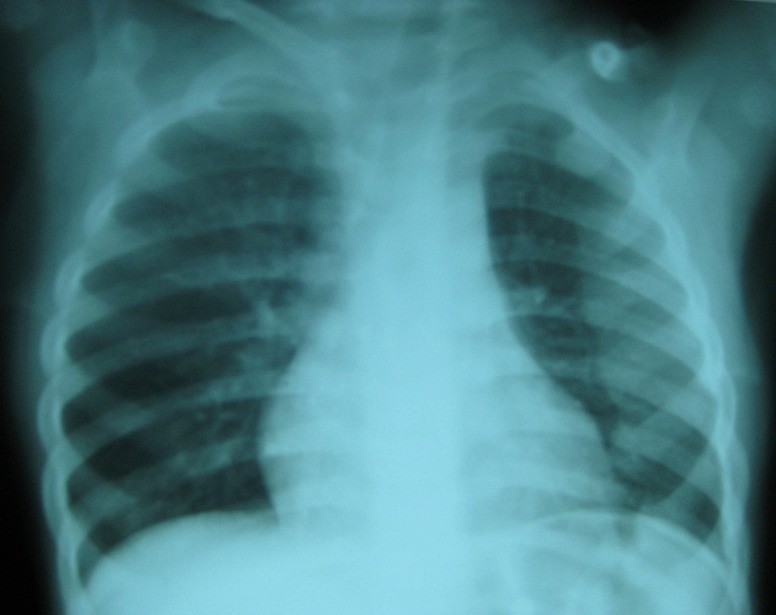
Radiographie du thorax de face du patient:déminéralisation des côtes avec élargissement en palette de l'extrémité antérieure des arcs costaux, mieux visible à gauche. Cal osseux médio-claviculaire gauche

**Figure 2:**
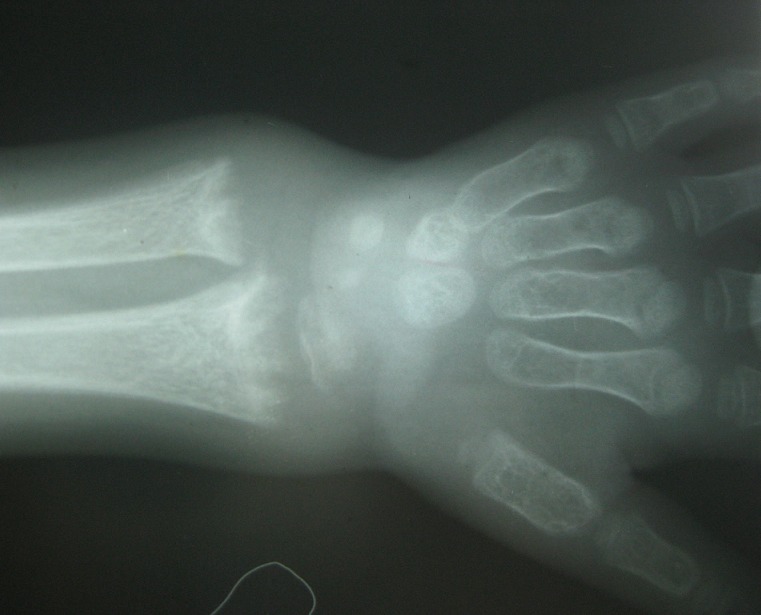
Radiographie du poignet montrant un élargissement en cupule des métaphyses dont les limites sont floues. Déminéralisation osseuse

Le scanner cérébral était normal, hormis la microcéphalie. Les radiographies osseuses montraient (Figure 1, Figure 2) une déminéralisation diffuse des côtes et des os longs, un chapelet costal, un élargissement transversal des poignets avec incurvation en cupule de la ligne métaphysaire, un cal osseux au niveau du tiers moyen de la clavicule gauche. Un électroencéphalogramme obtenu au 5^ème^ jour d'hospitalisation était normal.

Le patient recevait du valium intra rectal et du calcium intraveineux aux urgences ; puis une supplémentation en vitamine D (5000 U.I par jour) et du calcium per os étaient débutés. Le traitement par phénobarbital était poursuivi à la dose d'entretien de 5 mg/kg/j.

Le contrôle des convulsions était effectif dès le 3^ème^ jour d'hospitalisation. La supplémentation vitaminocalcique devait être poursuivie pendant au moins 6 mois. De même, l'apport en vitamine D devait être assuré tant que durait le traitement par phénobarbital.

## Discussion

L’épilepsie est une comorbidité assez fréquente chez les patients infirmes moteurs cérébraux [2]. Elle est habituellement lésionnelle, réfractaire et de sévérité corrélée au type de paralysie cérébrale, ou d'anomalies à l'imagerie cérébrale. La perte du contrôle des crises épileptiques dans ce contexte peut donc n’être que l'expression d'une pharmaco-résistance secondaire de l’épilepsie. Ce qui n’était pas le cas chez notre patient qui ne présentait aucune lésion potentiellement épileptogène au scanner cérébral.

La mauvaise observance au traitement est une autre cause majeure de reprise de crises, à rechercher par le clinicien. Le dosage du taux sanguin résiduel de l'antiépileptique permet de l'objectiver. Dans l'impossibilité d'obtenir une barbitémie dans notre contexte, l'interrogatoire des parents et le comptage des comprimés restants avaient été utilisés. Ces méthodes assez fiables [3], sont également utilisées dans le suivi de l'observance thérapeutique dans la tuberculose ou le VIH/SIDA dans notre milieu. Elles avaient permis de retrouver une bonne observance thérapeutique chez notre patient. Toutefois, la littérature rapporte malgré une bonne observance, des risques de perte du contrôle de l’épilepsie due au passage à une forme générique de l'antiépileptique en cours [4], ce qui n’était pas le cas chez notre patient.

Des causes de convulsions occasionnelles non fébriles devraient également être systématiquement recherchées, notamment des troubles hydroélectrolytiques et métaboliques. Dans notre cas, un rachitisme avec hypocalcémie avaient été décelés, ce qui nous avait permis de conclure à des convulsions hypocalcémiques.

En réalité, notre patient avait plusieurs raisons d'avoir une altération de la densité minérale osseuse et un risque accru de fractures. D'une part, l'absence de mise en charge en rapport avec son handicap, un risque plus élevé de chutes [5], et une déminéralisation généralisée possible du fait de sa mobilité réduite. En effet, notre patient était classé au niveau IV du système de classification de la fonction motrice globale de Palisano [1]. Bischof et al [6] rapportent d'ailleurs dans une étude publiée en 2002, une incidence élevée de fractures des os longs chez des patients avec paralysie cérébrale en institution. Un rachitisme ou une ostéomalacie avaient été mis en évidence chez eux.

D'autre part, la littérature rapporte une déminéralisation osseuse iatrogène imputée à la prise de certains antiépileptiques au long cours (barbituriques, phénytoïne, valproate de sodium, carbamazépine) [7]. Ces médications ont un effet sur l'activité microsomale hépatique (cytochrome P450) conduisant à une accélération du métabolisme de la vitamine D et sa conversion en métabolites biologiquement inactifs [8]. Une inhibition de l'expression de la 25-hydroxylase [9], une diminution de l'absorption intestinale du calcium, ou une augmentation de la résorption calcique osseuse sont des mécanismes également décrits.

Le risque de fractures est de deux à six fois plus élevé chez l’épileptique que dans la population générale [10] et concerne jusqu’à 20% de patients cérébrolésés à mobilité limitée au cours de leur vie [11]. Cependant, il est aussi admis que le handicap est un facteur de risque d'abus et de négligence chez l'enfant. Mais les fractures dans ce contexte concernent volontiers les os longs mais sont souvent d’âges différents [12]. Une maltraitance avait donc été évoquée par principe chez notre patient surtout en raison de son handicap, mais exclue devant le peu de consistance des arguments en faveur.

Le rachitisme aurait pu être décelé avant l'arrivée dans notre centre. En effet, l'affection peut être de découverte fortuite lors d'un examen systématique chez un patient apparemment en bonne santé ; au cours de la surveillance d'un traitement ou d'une affection susceptibles d'induire une déminéralisation osseuse ; devant toute fracture inexpliquée ou survenant pour un traumatisme minime. Il peut aussi se faire devant des atteintes squelettiques classiques ou à l'occasion de manifestations d'une hypocalcémie conséquente comme c’était le cas chez notre patient.

Toutefois, le rôle rachitigène d'une exposition insuffisante au soleil ne saurait être ignoré. Même dans notre milieu intertropical à l'ensoleillement optimal tout au long de l'année, le rachitisme est rapporté au sein de couches de populations qui pour des raisons religieuses ou culturelles, s'exposent peu au soleil. Par ailleurs, l'exposition au soleil du nourrisson ou du petit enfant est fortement dépendante de l'adulte. Il en est de même pour les sujets en situation de handicap avec mobilité réduite qui parfois font l'objet de surprotection ou de confinement par peur de stigmatisation.

## Conclusion

Le rachitisme peut être révélé par des convulsions hypocalcémiques. Il doit être exclu devant une perte du contrôle des crises chez les patients épileptiques à mobilité réduite avant toute escalade thérapeutique. Ces patients sous antiépileptique au long cours devraient bénéficier d'une surveillance de la densité osseuse, d'une exposition suffisante aux rayonnements solaires et en cas de besoin d'une supplémentation en vitamine D.

## References

[1] Palisano R, Rosenbaum P, Walter S, Russell D, Wood E, Galuppi B (1997). Le système de classification de la fonction motrice globale de la paralysie cérébrale. Dev Med Child Neurol..

[2] Hadjipanayis A, Hadjichristodoulou C, Yoroukos S (1997). Epilepsy in patients with cerebral palsy. Dev Med Child Neurol..

[3] Paschal AM, Hawley SR, St Romain T, Ablah E (2008). Measures of adherence to epilepsy treatment: Review of present practices and recommendations for future directions. Epilepsia..

[4] Berg MJ, Gross RA, Tomaszewski KJ, Zingaro WM, Haskins LS (2008). Generic substitution in the treatment of epilepsy: Case evidence of breakthrough seizures. Neurology..

[5] Leet AI, Mesfin A, Pichard C, Launay F, Brintzenhofeszoc K, Levey EB, Sponseller DP (2006). Fractures in children with cerebral palsy. J Pediatr Orthop..

[6] Bischof F, Basu D, Pettifor JM (2002). Pathological long-bone fractures in residents with cerebral palsy in a long-term care facility in South Africa. Dev Med Child Neurol..

[7] Samaniego EA, Sheth RD (2007). Bone consequences of epilepsy and antiepileptic medications. Semin Pediatr Neurol..

[8] Yamamoto T (2010). Secondary osteoporosis Update – Osteomalacia. Clin Calcium..

[9] Hosseinpour F, Ellfolk M, Norlin M, Wikvall K (2007). Phenobarbital suppresses vitamin D3 25-hydroxylase expression: a potential new mechanism for drug-induced osteomalacia. Biochem Biophys Res Commun..

[10] Nakken KO, Taubøll E (2010). Bone loss associated with use of antiepileptic drugs. Expert Opin Drug Saf..

[11] Sturm PF, Alman BA, Christie BL (1993). Femur fractures in institutionalized patients after hip spica immobilization. J Pediatr Orthop..

[12] Epelbaum C (1999). Maltraitance et sévices à enfant (hors abus sexuels). Encycl Méd Chir.

